# 

**Published:** 1823-01

**Authors:** 


### LONDON MEDICAL AND PHYSICAL JOURNAL.

CONTAINING

ORIGINAL CORRESPONDENCE OF EMINENT PRACTITIONERS,

AND

CRITICAL ANALYSIS OF NEW WORKS

RELATING TO MEDICINE, SURGERY, MIDWIFERY, CHEMISTRY, PHARMAC
BOTANY, AND NATURAL HISTORY.

---

EDITED BY

RODERICK MACLEOD, M.D.

LICENTIATE OF THE COLLEGE OF PHYSICIANS OF LONDON;
MEMBER OF THE MEDICO-CHIRURGICAL SOCIETY OF LONDON, AND OF THE
ROYAL MEDICAL SOCIETY OF EDINBURGH;
PHYSICIAN TO THE WESTMINSTER GENERAL DISPENSARY;
TO THE INFIRMARY FOR CHILDREN, AND TO THE SCOTTISH HOSPITAL:

AND

JOHN BACOT, Esq.

MEMBER OF THE ROYAL COLLEGE OF SURGEONS;
AND LATELY SURGEON TO HIS MAJESTY'S GRENADIER REGIMENT
OF FOOT GUARDS.

---

VOL. XLIX.
FROM JANUARY TO JUNE, 1823.

Et quoniam variant morbi, variabimus artes;

Mille mali species, mille salutis erunt.

Surgeon Genl's Office LIBRARY
45582

---

*LONDON:*

PRINTED FOR THE PROPRIETORS,
*By J. and C. Adlard, 23, Bartholomew Close;*
PUBLISHED BY J. SOUTER, 73, ST. PAUL'S CHURCH-YARD;
AND MAY BE HAD OF ALL BOOKSELLERS.

W1
L031

### MONTHLY CATALOGUE OF MEDICAL BOOKS.

*** *It having been hinted to us that gentlemen, sending copies of their Works, prefer having their titles given at length, it is our intention, hereafter, to comply with this suggestion: but it is to be observed, that no books can be entered on this list except those sent to us for the purpose;—in the lists hitherto transmitted, we find that the names of works have frequently been given as published, which have not appeared for weeks, or even months after.*

Practical Observations on the Treatment and Cure of several Varieties of Pulmonary Consumption, and on the Effects of the Vapour of Boiling Tar in that Disease. By Sir Alexander Crichton, M.D. F.R.S. Physician in ordinary to their Imperial Majesties the Emperor and Dowager Empress of Russia, and to his Royal Highness the Duke of Cambridge; Knight of the Red Eagle of Prussia, of the second Class, &c.

A System of Osteology; comprising the Bones and Articulations of the Human Skeleton: to which is added, a Description of the Organs of Sight and Hearing, and the Peculiarities of the Fœtus. The whole intended and arranged as an Appendix to the London Dissector, to supply its Deficiencies, and render it a complete Compendium of Anatomy.—London, 1822.

Illustrations of the Inquiry respecting Tuberculous Diseases. By John Baron, M.D. Physician to the General Infirmary at Gloucester.—London, 1822. pp. 233, with five coloured Plates.

Observations on the Acute and Chronic Dysentery of Ireland; containing an Historical View of the Progress of the Disease in Ireland, with an Inquiry into its Causes, and an Account of its Symptoms and Mode of Treatment; with a Report of selected Cases. By John O'Brien, M.D. Fellow and Censor to the King and Queen's College of Physicians in Ireland, and Physician to the Fever Hospital and House of Recovery, Cork-street, Dublin.—Dublin, 1822. pp. 93.

Pharmacopœia Imperialis, sive Pharmacopœiæ Londinensis, Edinburgensis, et Dublinensis; collatæ cum Notis Anglicis, Decompositiones Chemica exponentibus.—Londini, 1823. pp. 255.

---

### NOTICES TO CORRESPONDENTS.

*The Communications of* Mr. Bampfield, Mr. Armstrong, Mr. Aspray, Dr. Belcombe, Mr. Orton, Mr. Ernest, *and* Dr. Webster, *have been received.*

*While we have much gratification in acknowledging the receipt of so many Communications, we are compelled to apologize for the delay with which some may be apt to charge us in giving them to the public. This, however, has been unavoidable; established custom obliging us to substitute an Historical Essay of our own at the commencement of the volume, in place of the more valuable matter contained in the papers reserved for the ensuing Number.*

*We beg to inform our valued Correspondent,* Mr. Hutchinson, *of Southwell, that his papers were delayed, with the intention of placing them along with another Communication, tending to favour the views he has so ably supported. This Communication has not been received; but those of* Mr. H. *shall appear in our Number for February.*

